# Using Machine Learning to Diagnose Autism Based on Eye Tracking Technology

**DOI:** 10.3390/diagnostics15010066

**Published:** 2024-12-30

**Authors:** Ameera S. Jaradat, Mohammad Wedyan, Saja Alomari, Malek Mahmoud Barhoush

**Affiliations:** Computer Science Department, Yarmouk University, Irbid 21163, Jordan; ameera@yu.edu.jo (A.S.J.); sajaalomari1997@gmail.com (S.A.); malek@yu.edu.jo (M.M.B.)

**Keywords:** image classification, ASD diagnosis, image processing, machine learning, deep learning, hybrid learning, stacking ensemble learning, MobileNet

## Abstract

**Background/Objectives:** One of the key challenges in autism is early diagnosis. Early diagnosis leads to early interventions that improve the condition and not worsen autism in the future. Currently, autism diagnoses are based on monitoring by a doctor or specialist after the child reaches a certain age exceeding three years after the parents observe the child’s abnormal behavior. **Methods:** The paper aims to find another way to diagnose autism that is effective and earlier than traditional methods of diagnosis. Therefore, we used the Eye Gaze fixes map dataset and Eye Tracking Scanpath dataset (ETSDS) to diagnose Autistic Spectrum Disorder (ASDs), while a subset of the ETSDS was used to recognize autism scores. **Results:** The experimental results showed that the higher accuracy rate reached 96.1% and 98.0% for the hybrid model on Eye Gaze fixes map datasets and ETSDS, respectively. A higher accuracy rate was reached (98.1%) on the ETSDS used to recognize autism scores. Furthermore, the results showed the outperformer for the proposed method results compared to previous works. **Conclusions:** This confirms the effectiveness of using artificial intelligence techniques in diagnosing diseases in general and diagnosing autism, in addition to the need to increase research in the field of diagnosing diseases using advanced techniques.

## 1. Introduction

Autism is a developmental disorder that has many symptoms such as communication, behavior, and social interaction. It is called a spectrum disorder because it affects children varying degrees and differently, ranging from moderate to grave. Autism has recently spread around the world. Due to the variety of symptoms among autistic children, it is difficult to diagnose them. Autism is typically diagnosed by a group of specialists observing the child’s behavior, which takes a long time and carries the risk of misdiagnosis, because the behavior of an autistic child can resemble the behavior of other children with different psychological disorders. As a result, technical improvements were required to seek alternate diagnostic approaches [1,2,3].

Autism spectrum disorder varies greatly in symptoms and levels of severity, which vary from child to child. The child’s tendency to isolate and withdraw, the trouble with social communication, the difficulty with linguistic abilities, the difficulty with learning, and the lack of interest in games and colors, as is typical for children their age, are some of the most significant indicators that challenge autism children [3,4].

The prevalence of autism has seen a notable increase globally, with variations depending on diagnostic practices, awareness, and regional factors. Also, autism is more commonly diagnosed in males, with a ratio of approximately 4:1 compared to females. Early diagnosis of autism may be accurately identified in children as early as age three. Unfortunately, many do not acquire a diagnosis until afterwards. Achievements can be substantially improved by early intervention [1].

Autism involves a multi-step process that includes developmental screening, comprehensive diagnostic evaluation, and sometimes additional tests to rule out other conditions. For example, the Diagnostic and Statistical Manual of Mental Disorders, Fifth Edition (DSM-5) is a comprehensive classification method for mental disorders. It is widely used by clinicians and researchers for diagnosing and classifying mental health conditions, but DSM-5 has many limitations, such as clinicians should use it as a guide rather than a definitive authority, considering individual patient contexts and the potential for cultural and individual variability in symptoms and experiences [1,5].

Machine learning holds a significant promise for improving the diagnosis and treatment of autism. By leveraging large datasets and advanced algorithms, machine learning (ML) can enhance early detection, diagnostic accuracy, and personalized interventions. Machine learning has been used to diagnose autism, such as diagnosis through studying the movement [1] of the upper and the lower limbs, which includes the child reaching the walking stage, when approximately two years of age. The importance of our proposed study is that it relies on eye movement analysis and enables us to diagnose children with autism at a very early stage.

Given the necessity of early diagnosis, it was important to find computer-based methods and systems that would make detecting the autism spectrum faster, more accurate, and more timesaving for professionals to treat children [1,2]. One of the most recent ways of diagnosing autism and recognizing its severity is eye tracking monitoring. It is one of the most important vital signs for an autism diagnosis. Similar differences in visual patterns of gaze behavior and eye movement dynamics were seen between autistic and non-autistic children.

Eye tracking is one of the most important vital indicators for diagnosing autism spectrum disease, and early diagnosis of the disease can help evaluate rapid treatment methods to reduce symptoms, which can help the child to be more independent and better integrated into society. Therefore, the study aims to build a hybrid model based on MobileNet and Stacking Ensemble Learning to diagnose autistic and non-autistic children with the ability to recognize the autism degree, based on visual patterns of gaze behavior and eye movement dynamics that describe velocity and acceleration. The proposed system will contain several steps, which are used to get an accurate and real-time system.

In this research, we are taking advantage of the deep learning and machine learning models to build a hybrid model to provide a solution for an efficient and speedy ASD diagnosis. We employed the deep learning MobileNet model for feature extraction and used machine learning for dimensionality reduction using principal component analysis (PCA) and use a stacking ensemble learning based on combining support vector machine (SVM) and K-nearest neighbor (KNN) to classify it. Recently, hybrid learning showed more effectiveness than traditional machine learning approaches [4].

## 2. Literature Review

In this section, recent scientific studies have been presented on ASD diagnosis and recognizing ASD scores based on eye tracking using ML and deep learning (DL) models. This section is divided into ML and DL approaches. Numerous researchers have attempted to use eye tracking to observe and analyze the behavior of people with ASD.

Ref. [6] used four machine learning classifiers (random forest, SVM, decision tree, and linear discriminant analysis) based on eye tracking data from face-to-face conversations to gain a better result by combining features on the length of the conversation and visual fixation. The SVM achieved the most accurate results, reaching 92.31%. eye tracking

Ref. [7] used a k-means clustering model that split the eye tracking scan path dataset into four clusters and extracted the best result based on different classifiers (DT, GB, KNN, LR, MLP, NB, RF, SVM, and XGB). To achieve a better result, the data are processed using these steps (resizing the image to (128, 128) and converting it to grayscale). The Multi-Layer Perceptron (MLP) achieved the best result in Cluster 1, the accuracy reaching 87%.

Ref. [8] used a set of multivariate supervised machine learning (naïve Bayes, XGBoost, KNN, random forest, and SVM) for binary classification, based on the eye tracking paradigm in a virtual environment. The SVM achieved the best accuracy, reaching 86%. Ref. [9] used supports vector machines, based on the images of children, and used Viola–Jones to identify the eye, achieving an accuracy of 89%.

Ref. [10] used convolution neural network (CNN), based on the visual representation of eye tracking scanpaths. The model achieved 90% accuracy. Ref. [11] used two techniques. The first technique extracts features using PCA and three machine learning classifiers (BDT, DSVM, and DJ). The second technique extracts features using CNN and deep neural network (DNN) classifiers. To gain a better result, the data are processed using these steps (image augmentation, resizing the image, and converting it to grayscale). Based on the visual representation of eye tracking scanpaths, the deep neural network model outperformed typical machine learning.

Ref. [12] used CNN, based on the fixation maps of the corresponding observer’s gaze at a given image. The model achieved 75.23% accuracy for validation. Ref. [13] used four techniques for binary classification, based on the visual representation of eye tracking scanpaths. The first technique uses FFNNs and artificial neural network (ANN) and is based on feature classification extracted by a hybrid method (LBP) and (GLCM). The second technique is using pre-trained (CNN) models such as GoogleNet and ResNet-18. The third technique is the hybrid method (GoogleNet + SVM and ResNet-18 + SVM). The FFNNs and ANNs achieved the best accuracy, reaching 99.8%. However, the researchers adopted ResNet18 because it made lesser errors compared to the other models and achieved an accuracy reaching 97.6%. Table 1 is a summary of the literature review.

## 3. Methodology

**Figure 1:**
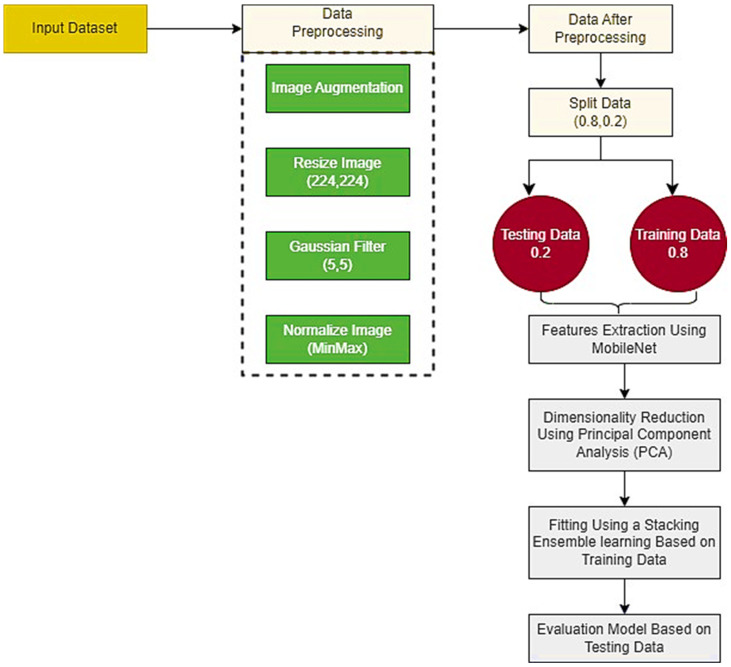
Overall research design.

This section explains the proposed methodology for diagnosing autism spectrum disease with the ability to recognize the degree of autism. The contribution of this work is to develop a hybrid model through which one can automatically diagnose autism spectrum disease with the ability to recognize the autism degree in the image that contains visual patterns of gaze behavior and eye movement dynamics data. We use image augmentation technique to increase the images and use more steps for image enhancement, including resizing images and removing noise using the Gaussian filter. We rely on deep learning, especially the MobileNet model, to extract features from images, principal component analysis for dimensionality reduction, and machine learning, especially stacking ensemble learning, for classification. Figure 1 shows the overall design of the proposed method.

### 3.1. Dataset

**Figure 2:**
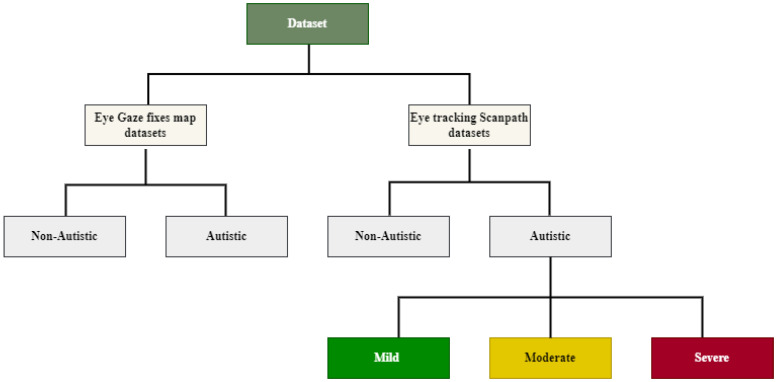
Dataset description.

Figure 2 shows the used dataset in this work, which contains two types of image datasets for ASD diagnosis and one type of image dataset to recognize the ASD score.

The first image datasets for ASD diagnosis are ETSDS, which contain visual patterns of gaze behavior and eye movement dynamics data. This dataset is available on the Figshare repository and contains images that contain visual patterns of gaze behavior and eye movement dynamics data of autistic and non-autistic children, and the Excel sheet contains some attributes about children, one of which is an autism score, published by [14], which contains 215 images of autistic children and 319 images of non-autistic children [15].

This paper symbolizes the dataset as follows:

1. Eye gaze fixes map datasets (1).
2. ETSDS (2).
3. ETSDS for recognizing ASD scores (3).

The movement dynamics were indicated by the color shift across the line. The RGB component values were tweaked using velocity, acceleration, and jerk with respect to time. For example, the values of velocity vary from black (low) to red (high). As a result, greater velocity readings eventually move towards deeper red values. Similarly, the acceleration and jerk were set using green and blue color gradients, respectively [14].

**Figure 3:**
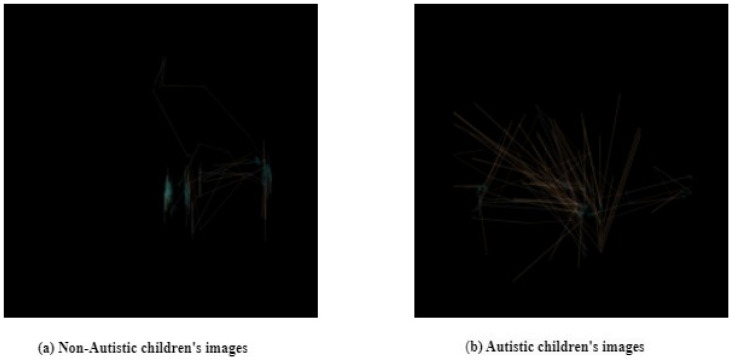
ETSDS.

Figure 3 shows the eye tracking scanpaths as a visual representation of a child that does not have an autism spectrum disorder. Also, it shows the eye tracking scan path as a visual representation of a child with autism spectrum disorder.

The second image dataset for ASD diagnosis is eye gaze fixing the map datasets, which contain a fixed map of gaze behavior. This dataset is available on the Zenodo repository and contains 300 images of autistic children and 300 images of non-autistic children.

**Figure 4:**
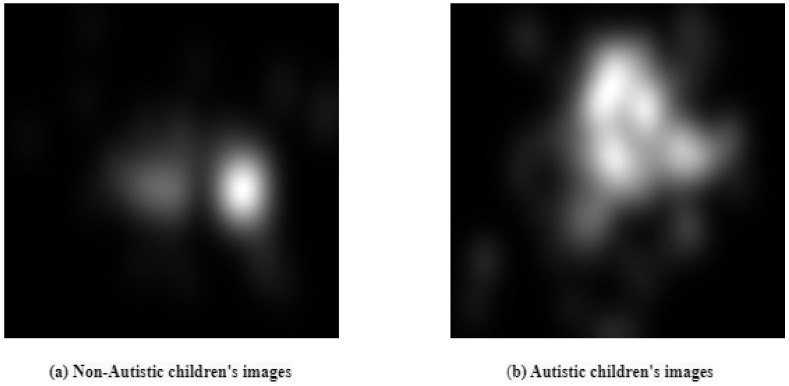
Eye gaze fixes map datasets.

Figure 4 shows the eye gaze fixed map of a child not infected with an autism spectrum disorder. Also, it shows the eye gaze fixed map of a child with an autism spectrum disorder.

The third image datasets for recognizing ASD scores are a subset of ETSDS that contain visual patterns of gaze behavior and eye movement dynamics data. The dataset contains nearly 98 images that are mild scores, 87 images that are moderate scores, and 34 images that are severe scores, which were separated manually.

**Figure 5:**
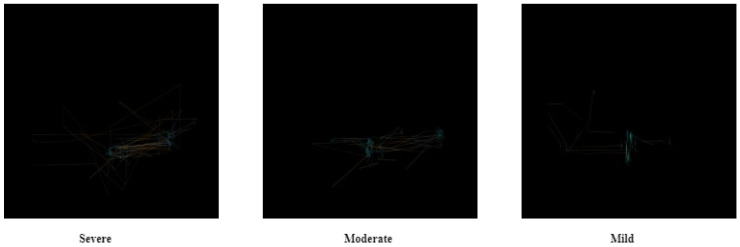
ETSDS for recognizing ASD scores.

Figure 5 shows ETSDS for recognizing ASD scores with three cases. All these cases are individuals with autism but with different severities.

### 3.2. Data Preprocessing

Preprocessing is a critical phase in image classification, where it is used to reduce problems in images. The data are prepared to be suitable for deep and machine learning by extracting and understanding information from used images in an accurate technique. The quality of the photos used affected the model’s performance, and the higher the quality of the images, the better the model’s performance.

Image Augmentation

To have successful DL models, data augmentation (DA) is considered a key element, leading to better accurate prediction values, especially when using datasets with large sizes [16]. In addition, training data are generated by using augmentation techniques by performing changes to the images, such as zooming, rotation, shearing, and flipping.

Data augmentation by data variety can improve the deep learning model performance and play a main role in minimizing the problem of overfitting [17]. Data deficiency is one of the most common problems in medical problems in deep learning. This issue can be managed by data augmentation, which has been used in many studies to increase images before they were included in the model, such as [18].

Data augmentation by data variety can improve the deep learning model performance and play a main role in minimizing the problem of overfitting [19], which used image augmentation techniques to detect COVID-19 by increasing the size of the images for the detection of COVID-19. The CNN model achieved 85% accuracy before data augmentation and achieved the best accuracy, reaching 95% after data augmentation, using augmentation techniques to increase the prostate diffusion-weighted magnetic resonance imaging training dataset [20], as well as it was used to increase Breast Cancer Microscope Image, which could be improved by applying data augmentation [21], and in brain tumor detection [22].

In this work, the augmentation techniques generated used three augmentation techniques (random rotation, horizontal flip, and vertical flip).

- Random rotation

The process of random rotation augmentation involves randomly selecting the rotation angle that can be positive or negative, applying that rotation to the image. Angle 15 was selected for this work.

- Horizontal flip

The process of horizontal flip augmentation is done by horizontal flipping along the vertical axis of the image, providing a mirror image of the original image and reversing the order of the image columns or pixels.

- Vertical flip

Augmentation of a vertical flip involves the image flipping vertically along the horizontal axis in order to reverse the order of rows or pixels along the horizontal axis.

Table 2 shows the effect of augmentation techniques on increasing the number of non-autistic and autistic children’s images and autism score images.

- Resize Images

In preparing an image dataset for a machine learning classification, resizing an image is an essential step and that standardization of the image size can enhance the machine learning model accuracy and efficiency, providing faster training. To improve the model’s detection features and patterns, training on images with consistent size ability is used. The image was resized into (224, 224) in this work, for width and height to extract the features in proportion to the model used.

- Removing noise using Gaussian filter

Removing noise from images can improve the performance of machine learning models by making it easier for them to extract useful features from the image. The presence of noise in the dataset significantly reduced the classification accuracy and poor prediction results [23]. Noisy images can make it difficult for machine learning models to accurately identify patterns and features in the image. By removing noise, we can improve the quality of the image, making it easier for the model to obtain an accurate and useful outcome [24]. The Gaussian blur filter has been used in many studies to enhance images before they were included in the model, such as [25], where they obtained better results when they used it with a machine learning algorithm.

**Figure 6:**
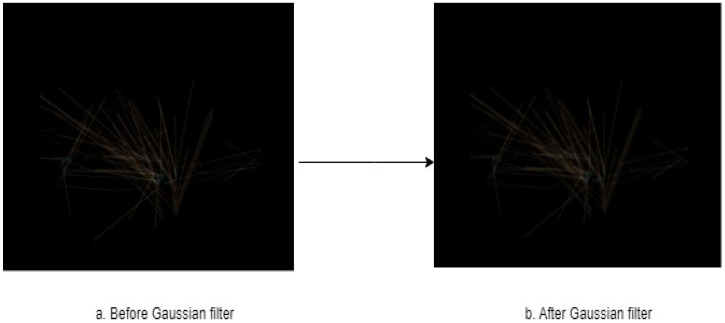
Image after and before using the Gaussian filter.

From several techniques that can be used to remove noise from images, in our research, we use a Gaussian filter. Gaussian blur is a typical image processing method used to remove noise and smooth out features in an image. When Gaussian blur is applied to an image, each pixel is replaced with a weighted average of its nearby pixels. Gaussian is based on assigning a weighted average to neighboring pixels, depending on the Gaussian (Normal) distribution. This averaging method aids in the reduction of high-frequency noise and rapid changes in the image, resulting in a smoother appearance. The size of the Gaussian filter, which is commonly set by the standard deviation (sigma) parameter, controls the degree of blur applied. A greater sigma value results in a broader Gaussian distribution and more blurring. In this work, we use a (5, 5) sigma. Figure 6 shows the image after and before using the Gaussian filter.

Normalize images

Normalizing images is the process of transforming an image’s pixel values to a specified range to facilitate feature extraction and model training, is a common step in image processing and computer vision tasks to obtain a better performance from models.

In this work, we used min–max normalization techniques that scale pixel values to a specific range, often between 0 and 1, and normalize each pixel value by subtracting the minimum and dividing by the range (maximum minus minimum), can be calculated using the formula in Equation (1):

(1) $$X_{normaliz}=\frac{\left(X-X_{minimum}\right)}{{X_{maximum}-X}_{minimum}}$$

Equation (1) is the min–max normalization equation.

### 3.3. Splitting Data

Splitting data is an important stage in machine learning (ML) to create separate subsets of datasets by dividing it into two partitions: the training set and the testing set. Where the training set is the greatest percentage of the dataset used to train the model, and the testing set is used to evaluate the final performance of the trained model, as it contains unseen data that the model was not exposed to during training.

The most common split ratios are 70:30 or 80:20, indicating the percentage of data allocated to the training and test sets, respectively. In this work, the dataset was divided into 80% for the training model and 20% for the testing model, because they are some of the best-split ratios [26].

### 3.4. Features Extraction Using MobileNet

Feature extraction is an important stage in machine learning; it is the process of extracting significant and useful features from the training and testing sets, which lead to representing the data with a smaller set of features, allowing for the building of an effective and accurate model, improved model performance, and reduced storage requirements.

There are several ways for feature extraction, some of which are classic and others that are using deep learning models. This work uses deep learning models to extract features due to their efficiency and high accuracy. We used the MobileNet model, which was previously explained in the first section.

MobileNet is pre-trained on the ImageNet dataset. In this work, we use it to extract features from various datasets; therefore, we fine-tune the model by removing the top layers; to do so, set the included top argument to false when loading a MobileNet model, and set the trainable parameter to False. The last fully connected (FC) layers will not be loaded, allowing access to the intermediate features extracted by the convolutional layers.

The extracted features are represented by patterns and color gradients. These gradients, which were previously explained, represent speed and acceleration, while the pattern represents the movement of the child’s eye. From ETSDS for recognizing ASD scores and ETSDS, we extracted patterns and color gradients. While eye gaze fixes map datasets, we extracted just patterns, because it does not contain colors representing speed and acceleration.

### 3.5. Dimensionality Reduction Using PCA

Principal component analysis can be used as a dimensionality reduction technique. It aids in transforming high-dimensional features into a lower-dimensional representation while retaining the most important features.

By reducing the dimensionality of the data, PCA can simplify complex datasets, remove redundant or noisy features, and improve computational efficiency and accuracy in subsequent machine learning tasks [27]. Such as [28], we built a hybrid model from PCA and ANN for Malware traffic classification, achieving high accuracy of about 99%.

**Figure 7:**
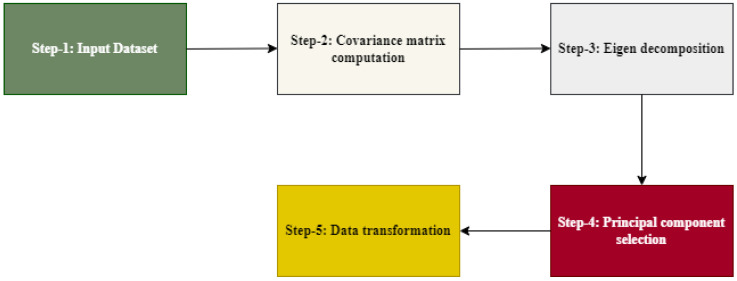
The principal component analysis work.

In this work, we selected 250 principal components from features. Figure 7 shows the concept of PCA works, where:

- Step 1 is the input dataset.
- Step 2 is the Covariance matrix computation, which represents the relationships and variances between different features.
- Step 3 is the Eigende composition, which involves obtaining the eigenvalues and eigenvectors by diagonalizing the covariance matrix. The eigenvalues reflect the amount of variance explained by each principal component, while the accompanying eigenvectors describe the feature space directions. Then, the eigenvectors associated with the highest eigenvalues (the largest variances) are selected as the principal components.
- Step 4 is the principal component selection, in which the number of principal components chosen depends on the desired dimensionality of the reduced data.
- Step 5 is data transformation, in which the original features are projected onto the selected principal components to obtain the lower-dimensional representation. This is done by taking the dot product between the original features and the eigenvectors.

### 3.6. Classification Using Stacking Ensemble Learning

Classification is a fundamental process in supervised machine learning that involves classifying data into classes based on its properties. There is a wide range of techniques for classification, and the techniques used are determined by the properties of the data, the complexity of the problem, and the required performance.

**Figure 8:**
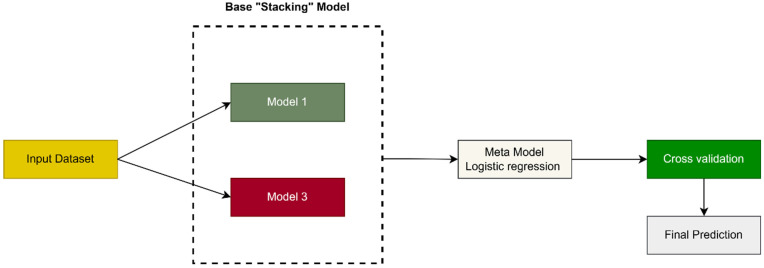
Stacking ensemble learning work in this work.

In this work, we use stacking ensemble learning, which was previously explained in the first section. We fine-tuned the base model, meta model, and cross-validation. Figure 8 shows stacking ensemble learning work. The model is trained using the training data before being evaluated on test data.

Base Model

The base model consists of two machine learning models, which are

Support Vector Machines (SVM)

In this work, we use the SVM model, which was previously explained in the first section. We fine-tuned some parameters, such as the kernel function, gamma parameter, degree, and random state, to improve the performance of the SVM classifier. The parameters can be adjusted to control the behavior and performance of the model. Table 3 shows the value for the parameters used in this work.

K-nearest neighbor (KNN)

In this work, we use the K-nearest neighbor (KNN) model, which was previously explained in the first section. We fine-tune the K parameter on 3.

Meta Model

The meta-model is logistic regression, which was previously explained in the first section.

Cross-Validation

Cross-validation is a technique used in machine learning to evaluate the performance of the model, which involves dividing the input data into subgroups used for training and evaluating the model on different combinations of these subsets. One of the most important reasons for overfitting and poor accuracy is the small dataset, which is the main objective of cross-validation to avoid this problem [29].

**Figure 9:**
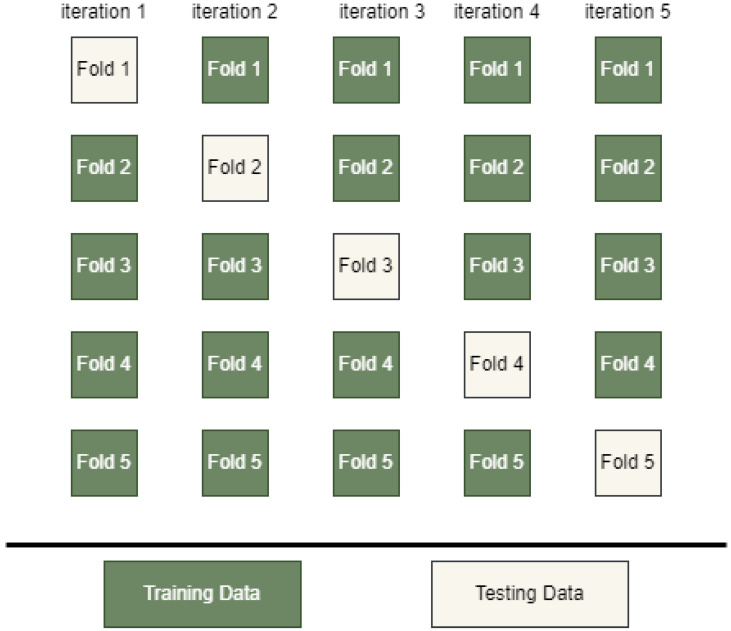
K-Fold cross-validation work.

In this work, we use K-fold cross-validation techniques to optimize stacked ensemble learning, which includes partitioning the training data into several parts, called K-fold, and used these data to train and evaluate the model simultaneously. In each iteration, one part is selected for model evaluation (testing), and the remaining parts are used for training. A good choice of k results in improved accuracy, but an incorrect selection of k may influence the model’s performance; therefore, after several experiments, we adopted the value 5 for the K-fold (20%). Figure 9 shows the K-fold cross-validation work.

K-fold cross-validation has been used in many studies to enhance the model performance before they were included in the proposed model, like [30,31], where they obtained better results when they used it with a machine learning algorithm.

## 4. Data Analysis and Interpretation

Datasets are used to train AI models, which are separated into two parts: training and testing. The training data are used for training and building the proposed method. The test data are used to evaluate the performance of models. This study’s data were separated into 80% for training and 20% for testing.

In this work, we used a confusion matrix to evaluate the model’s performance. The confusion matrix is a table used for evaluating the effectiveness of a classification model. It presents a detailed overview of the model’s predictions by comparing them to the dataset’s actual class labels. The matrix consists of four main components:

- True Positives (TP): The number of instances correctly predicted as positive.
- True Negatives (TN): The number of instances correctly predicted as negative.
- False Positives (FP): The number of instances incorrectly predicted as positive.
- False Negatives (FN): The number of instances incorrectly predicted as negative.
- Using these components, the performances of artificial intelligence models such as Accuracy, Specificity, Precision, and Sensitivity are extracted.

Accuracy: The percentage of correct predictions (true positive and true negative) divided by the total number of predictions can be calculated using the formula given by Equation (2):

(2) $$Accuracy=\frac{TP+TN}{TP+TN+FP+FN}$$

Equation (2) Accuracy Equation

Sensitivity: The percentage of correctly predicted positives (true positives) divided by the total number of positives predicted (true positives and false negatives) can be calculated using the formula given by Equation (3):

(3) $$Sensitivity=\frac{TP}{TP+FN}$$

Equation (3) Sensitivity Equation

Specificity: The percentage of correctly predicted negatives (true negatives) divided by the total number of negatives predicted (true negatives and false positives) can be calculated using the formula in Equation (4):

(4) $$Specificity=\frac{TN}{TN+FP}$$

Equation (4) Specificity Equation

Precision: The percentage of predictions positive to all cases, can be calculated using the formula given by Equation (5):

(5) $$Precision=\frac{TP}{TP+FP}$$

Equation (5) Precision Equation

### Research Tools

The research tool used in this research is Spyder Community Edition for implementing the proposed method Python code. We used machine learning libraries such as Sklearn, TensorFlow, CV2, and Matplotlib.

## 5. Experimental Results and Discussion

The evaluation results of the diagnosis are presented in this section of autism spectrum disease and recognize scores for autism based on the hybrid model (MobileNet and stacking ensemble learning). We used the MobileNet model to extract features from images and stacking ensemble learning to classify them. The experimental results for the proposed method are presented in this section. Also, this section shows the results of the proposed method depending on ETSDS, Eye Gaze fixes map datasets, and ETSDS for recognizing ASD scores.

As shown in Table 4, after several experiments, the proposed method using the hybrid model (MobileNet and stacking ensemble learning) with the different datasets achieves a high and consistent accuracy rate, where the optimal accuracy for ETSDS for recognizing ASD scores in accuracy reached approximately 98.18% with a sensitivity reached 98.08% and specificity of 99.03%. This was followed by ETSDS, as the accuracy reached approximately 98.02% and sensitivity reached 98.25% and specificity reached 97.82%. Eye Gaze fixes map datasets eventually came next in terms of accuracy, reaching approximately 96.17% with a sensitivity that reached 96.55% and a specificity that reached 95.83%.

We compare the proposed model with other models, and the results show the various pre-trained models used to extract features from all the datasets we used for training stacking ensemble learning. The following models were used: InceptionV3, Xception, VGG19, and MobileNet V1. In the MobileNet V1 model, the model performed best in terms of testing accuracy, precision, sensitivity, and specificity.

As Table 5 illustrates, the results were achieved while training the stacking ensemble learning model with features from various pre-trained models. The MobileNet V1 model achieved the best performance with all the datasets we used, followed by VGG19, InceptionV3, and Xception eventually came last. which is due to the different structures of each of them.

The results show the different results on all the datasets we used for applying the different cross-validation approaches in stacking ensemble learning with the value of k-fold changing each time. The following values were used: 5, 10, and 15. In k-fold equal 5, the model performed best in terms of testing precision, accuracy, specificity, and sensitivity.

As shown in Table 6, the results were achieved while training the stacking ensemble learning model with various k-fold values. We have adopted k-fold of 5. Despite the other k-fold values achieving good results, the k-fold is 5 value achieved consistent results with all the datasets we used.

This section shows the different results on all the datasets we used for applying the K-nearest neighbor (KNN) model in stacking ensemble learning while changing the value of K each time. The following values were used: 3, 5, and 10. In k equals 3, the model performed best in terms of testing precision, accuracy, specificity, and sensitivity. As shown in Table 7, the results were achieved while training the stacking ensemble learning model with various k values. We have adopted k equal 5. Despite the other values achieving good results, the k equal 5 value achieved consistent results with all the datasets we used.

The different results on all the datasets we used for applying the SVM model in stacking ensemble learning while we kept changing the value of the coef0 each time. The following values were used: default (0.0), 1.5, and 2.5. In coef0 equal to 1.5, the model performed best in terms of testing the precision, accuracy, specificity, and sensitivity.

As shown in Table 8, the results were achieved while training the stacking ensemble learning model with various coef0 values. We adopted coef0 equal to 1.5. Despite the other coef0 values achieving good results, the coef0 equal 5 value achieved consistent results with all the datasets we used.

The different results on all the datasets we used for applying in stacking ensemble learning with augmentation images and without augmentation images. The model performed best in terms of testing the precision, accuracy, specificity, and sensitivity.

As shown in Table 9, the results were achieved while training the stacking ensemble learning model with and without augmentation images. We adopted augmentation images and achieved consistent results with all the datasets we used.

### 5.1. Comparison of the MobileNet-SVM and the MobileNet-KNN Versus the MobileNet-Stacking Ensemble Learning on All the Datasets

This section shows the results of the proposed method as a hybrid model (MobileNet and stacking ensemble learning) comparison of MobileNet-SVM and MobileNet-KNN, where stacking ensemble learning is combined with SVM and KNN. We achieved this result by applying these models on ETSDS, Eye Gaze fixes map datasets, and ETSDS for recognizing ASD scores. The model performed best in terms of testing the precision, accuracy, specificity, and sensitivity.

As shown in Table 10, the results were achieved while training the models. We adopted MobileNet and stacking ensemble learning, because they worked to improve the results as expected.

### 5.2. Discussion

**Figure 10:**
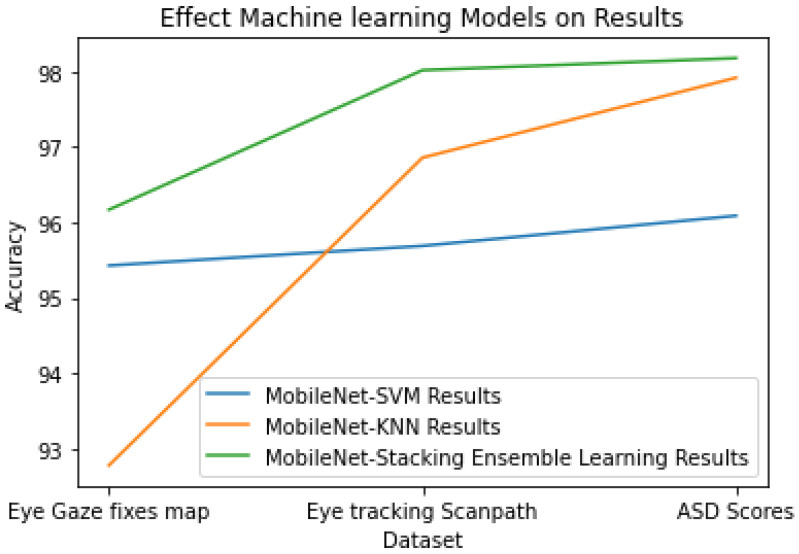
Effect of the machine learning models on the results.

In this study, we proposed a method using the hybrid model (MobileNet and stacking ensemble learning), with stacking ensemble learning and a combination of SVM and KNN to diagnose ASD from eye tracking, our proposed stacking ensemble learning outperformed the other common models. The results showed that using stacking ensemble learning in a combination of SVM and KNN instead of using SVM alone or using KNN alone performs better, and we can notice from Figure 10 the differences between the model results.

**Figure 11:**
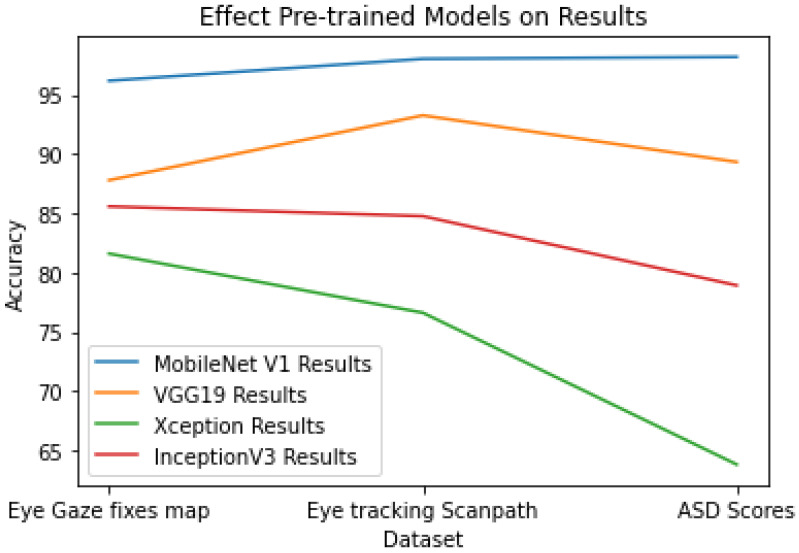
Effect of the pre-trained models on the results.

To obtain these results, we used various pre-trained models to extract features (InceptionV3, Xception, VGG19, and MobileNet V1). The results showed that using MobileNet V1 performs better, as Figure 11 shows the differences between the model results.

**Figure 12:**
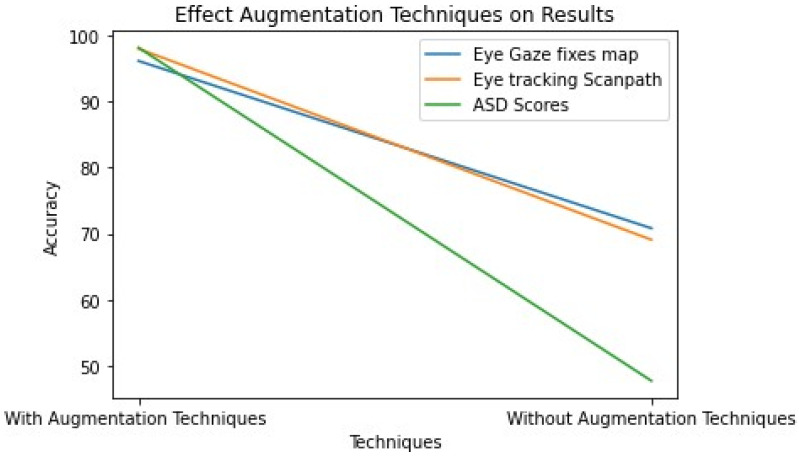
Effect of the augmentation techniques on the results.

Also, we used augmentation techniques to increase the size of the datasets to obtain better performance. The results showed that using augmentation techniques performs better, as Figure 12 shows the differences between the model results on datasets.

The results of the proposed method were compared with other studies that used the Eye Gaze fixes map dataset, ETSDS, and Eye tracking scan path ASD scores dataset for the diagnosis of ASD. Whereas, in the [13] study, the authors used ResNet18 for the diagnosis of ASD from the ETSDS, while, in the [12] study, the authors used CNN for the Eye Gaze fixes map dataset, and in [14], the authors used ANN for the diagnosis of ASD and recognize scores from the ETSDS. Table 11 shows a comparison result between the proposed method and previous studies that used the Eye Gaze fixes map dataset, ETSDS, and Eye Tracking scan path ASD scores dataset.

From Table 11, we can notice the preference for the hybrid model (MobileNet and stacking ensemble learning) compared to the ResNet18 model, the CNN, and the ANN, where the accuracy for the ResNet18 model in the [13] study reached 97.6%, with 75.23% for the CNN in the [12] study, and ≈83.0% for the ANN with Eye Tracking Scanpath ASD scores dataset and ≈90.0% with ETSDS in the [14] study. Finally, the accuracy rate for the hybrid model (MobileNet and stacking ensemble learning) in the proposed method reached 96.1% for Eye Gaze fixes map dataset, 98.0% for ETSDS, and 98.1% for the Eye Tracking Scanpath ASD scores dataset.

## 6. Conclusions

Autism has recently become more prevalent globally. Diagnosing autistic children is difficult because of their wide variety of symptoms. Due to the behavioral similarities between children with autism and those with other psychological disorders, autism is typically diagnosed by a team of specialists who evaluate the child’s behavior. This procedure is labor-intensive and offers a significant risk of misdiagnosis. As a result, technical improvements were required to seek alternate diagnostic approaches. Recently, scientists discovered that eye movement in children is a vital indicator for autism diagnosis.

Sometimes, it is challenging for specialists to diagnose autism due to the variability of symptoms among autistic children. Artificial intelligence technology helps in diagnosing diseases. Researchers have been drawn to the idea of diagnosing autism through tracking eye movement.

In this study, the primary concept behind MobileNet involves minimizing the parameters and operations while still achieving high accuracy in AI tasks. This division reduces computational complexity and model size while preserving the essential features. Stacking ensemble learning is a combination of SVM and KNN that helps achieve better performance. The proposed method used various eye tracking datasets for the experiments to show the effectiveness of the proposed methodology; in addition, the method used various eye tracking datasets for the experiments to show the effectiveness of the proposed methodology. The first dataset is the Eye Gaze fixes map, which consists of 300 images for autistic children and 300 for non-autistic children. The second dataset is the Eye Tracking Scanpath, which consists of 215 images for autistic children, and for non-autistic children 319 images were used. The third dataset is the subset of Eye Tracking Scanpath used to recognize autism scores, which consists of 215 images for severe autism scores images.

The experimental results using the hybrid model showed results that were convergent with all the datasets we used, where accuracy reached 96.1% with the Eye Gaze fixes map dataset, 98.0% for the ETSDS, and 98.1% for the Eye Tracking scan path ASD scores dataset. The hybrid model could accelerate diagnose early autism, facilitating timely interventions for improving developmental outcomes. Research suggests that early detection may enhance the effectiveness of the treatments.

## Figures and Tables

**Table 1:** Summary of the literature review.

	No. of Sample /Dataset	Purpose	Method	Performance
[14]	The visualization eye tracking scanpaths images dataset contains 547 images. Specifically, 328 images for the non-ASD participants, and 219 images for ASD-diagnosed. The dataset was augmented with an additional 2735 samples, where five synthetic images were generated for each visualization.	This study aimed to transforming eye tracking scanpaths into a visual representation, and ASD diagnosis using Machine learning.	ANN	ASD diagnosis: Accuracy = 90% Recognize autism scores: Accuracy = 83%
[6]	Eye tracking data from face-to-face conversations, where 20 children with TD and 19 children with ASD.	This study aimed to see if eye tracking data from face-to-face conversations could be used to differentiate between children with ASD and those with usual development (TD). They then tested if adding characteristics based on visual fixation and conversation time would improve classification performance.	SVM	Accuracy = 92.31% Specificity = 100% Sensitivity = 84.21%
[7]	The visualization eye tracking scanpaths images dataset contains 547 images. Specifically, 328 images for the non-ASD participants, and 219 images for ASD-diagnosed.	This study aimed to investigate eye-gazing images and identify autism by applying various machine learning techniques.	MLP	Accuracy = 87% Sensitivity = 88% Specificity = 69%
[10]	The scan path images contain 547 images (328 for non-ASD and 219 for ASD), the dataset was augmented with an additional 2735 samples.	This study aims to integrate eye tracking with visualization and machine learning.	CNN	Acc: 90% Sensitivity: 83%Precision: 80%
[8]	Eye gaze data represented by eye tracking paradigm in a virtual environment. 55 children participated, where 20 TD children and 35 ASD children.	This study aimed to distinguish between autistic and typically developing children in visual attention behaviors through an eye tracking paradigm in a virtual environment as a measure of attunement to, and extraction of, socially relevant information.	SVM	Accuracy = 86% Sensitivity = 91%
[9]	Images of children	This study aimed to build a program for diagnosing autism based on the eye from children’s images.	SVM	Accuracy = 89%
[11]	The visualization eye tracking scanpaths images dataset contains 547 images. Specifically, 328 images for the non-ASD participants, and 219 images for ASD-diagnosed. The dataset was augmented with an additional 2566 samples, 1519 images for the non-ASD participants and 1041 images for ASD-diagnosed.	This study aimed to investigate the effectiveness of various machine learning techniques to discover the best model for predicting autism using visualized eye tracking scan path images.	DNN	Sensitivity = 93.28% Specificity = 91.38%
[12]	Used the Fixation maps dataset contains 300 ASD fixation maps and 300 TD fixation maps.	This study aimed to observe whether eye tracking data of fixation maps could classify children with ASD and typical development (TD).	CNN	Accuracy = 75.23%
[13]	The visualization eye tracking scanpaths images dataset contains 547 images. Specifically, 328 images for the non-ASD participants and 219 images for ASD-diagnosed. The dataset was augmented, 1834 images for the non-ASD (training and validation) and 1750 images for ASD-diagnosed (training and validation).	This study aimed to develop three artificial intelligence techniques to detect autism spectrum disorder (ASD).	ResNet18	Accuracy = 97.6% Precision= 97.5% Sensitivity = 97% Specificity = 97%

**Table 2:** Number of images before and after augmentation.

Dataset	Before Augmentation	After Augmentation
Eye Tracking Scanpath Autistic	215	1963
Eye Tracking Scanpath Non-Autistic	319	1798
Eye Gaze Autistic	300	1397
Eye Gaze Non-Autistic	300	1394
Mild autism score images	98	652
Moderate autism score images	87	676
Severe autism score images	34	369

**Table 3:** Parameters setting for SVM.

Parameters	Value
kernel	ploy
Coef0	1.5
Degree	4
Random state	42

**Table 4:** Proposed method results.

Dataset	Accuracy	Precision	Sensitivity	Specificity
(1)	96.1%	96.9%	96.5%	95.8%
(2)	98.0%	98.4%	98.2%	97.8%
(3)	98.1%	98.2%	98.0%	99.0%

**Table 5:** Pre-trained model results with the stacking ensemble learning model.

Models	Dataset	Accuracy	Precision	Sensitivity	Specificity
InceptionV3	(1)	85.5%	89.2%	88.7%	82.7%
	(2)	84.7%	87.4%	86.2%	83.4%
	(3)	78.9%	78.9%	78.3%	87.9%
Xception	(1)	81.5%	83.4%	81.8%	81.3%
	(2)	76.6%	80.2%	79.0%	74.5%
	(3)	63.8%	63.6%	61.5%	77.3%
VGG19	(1)	87.7%	89.0%	87.7%	87.7%
	(2)	93.2%	95.4%	95.0%	91.7%
	(3)	89.3%	89.4%	89.2%	94.1%
MobileNet V1	(1)	96.1%	96.9%	96.5%	95.8%
	(2)	98.0%	98.4%	98.2%	97.8%
	(3)	98.1%	98.2%	98.0%	99.0%

**Table 6:** Cross-validations results with the stacking ensemble learning model.

Dataset	K-Fold	Accuracy	Precision	Sensitivity	Specificity
	5	96.1%	96.9%	96.5%	95.8%
(1)	10	96.3%	96.6%	96.2%	96.3%
	15	96.3%	96.6%	96.2%	96.3%
	5	98.0%	98.4%	98.2%	97.8%
(2)	10	97.6%	98.2%	98.0%	97.3%
	15	97.7%	98.2%	98.0%	97.6%
	5	98.1%	98.2%	98.0%	99.0%
(3)	10	98.1%	98.2%	98.0%	99.0%
	15	98.1%	98.2%	98.0%	99.0%

**Table 7:** Effect of the K on the results.

Dataset	K	Accuracy	Precision	Sensitivity	Specificity
	3	96.1%	96.9%	96.5%	95.8%
(1)	5	95.2%	96.3%	95.9%	94.7%
	7	95.4%	96.0%	95.6%	95.2%
	3	98.0%	98.4%	98.2%	97.8%
(2)	5	96.5%	96.7%	96.2%	96.7%
	7	95.9%	95.8%	95.2%	96.5%
	3	98.1%	98.2%	98.0%	99.0%
(3)	5	96.6%	96.6%	96.1%	98.1%
	7	96.0%	96.1%	95.5%	97.8%

**Table 8:** Effect of the coef0 on the results.

Dataset	coef0	Accuracy	Precision	Sensitivity	Specificity
	Default (0.0)	95.2%	96.3%	95.9%	94.7%
(1)	1.5	96.1%	96.9%	96.5%	95.8%
	2.5	95.8%	96.8%	96.5%	95.2%
	Default (0.0)	97.4%	98.0%	97.7%	97.1%
(2)	1.5	98.0%	98.4%	98.2%	97.8%
	2.5	97.7%	98.2%	98.0%	97.6%
	Default (0.0)	98.1%	98.1%	98.4%	99.0%
(3)	1.5	98.1%	98.2%	98.0%	99.0%
	2.5	97.9%	97.9%	97.8%	98.9%

**Table 9:** Effect of the augmentation images on the results.

Dataset	Cases	Accuracy	Precision	Sensitivity	Specificity
(1)	With Augmentation	96.1%	96.9%	96.5%	95.8%
	Without Augmentation	70.8%	72.8%	72.4%	69.3%
(2)	With Augmentation	98.0%	98.4%	98.2%	97.8%
	Without Augmentation	69.0%	82.7%	91.2%	45.2%
(3)	With Augmentation	98.1%	98.2%	98.0%	99.0%
	Without Augmentation	47.7%	41.5%	37.4%	65.2%

**Table 10:** The results of MobileNet-SVM, the MobileNet-KNN, and the MobileNet-Stacking Ensemble Learning on all the datasets.

Models	Dataset	Accuracy	Precision	Sensitivity	Specificity
MobileNet-SVM	(1)	95.4%	96.0%	95.6%	95.2%
	(2)	95.6%	95.6%	95.0%	96.3%
	(3)	96.0%	96.1%	95.5%	97.8%
MobileNet-KNN	(1)	92.7%	94.5%	94.0%	91.6%
	(2)	96.8%	96.8%	96.5%	97.1%
	(3)	97.9%	97.9%	98.2%	98.9%
MobileNet-Stacking Ensemble Learning	(1)	96.1%	96.9%	96.5%	95.8%
	(2)	98.0%	98.4%	98.2%	97.8%
	(3)	98.1%	98.2%	98.0%	99.0%

**Table 11:** Comparing the results with other works.

Study	Model	Dataset	Accuracy
[13]	ResNet18	ETSDS	97.6%
[12]	CNN	Eye Gaze fixes map dataset	75.2%
[14]	ANN	Eye Tracking scan path ASD scores dataset	≈83.0%
		ETSDS	≈90.0%
Proposed method	Hybrid Model (MobileNet and stacking ensemble learning)	ETSDS	98.0%
Proposed method	Hybrid Model (MobileNet and stacking ensemble learning)	Eye Gaze fixes map dataset	96.1%
Proposed method	Hybrid Model (MobileNet and stacking ensemble learning)	Eye Tracking scan path ASD scores dataset	98.1%

## Data Availability

The original contributions presented in this study are included in this article, and further inquiries can be directed to the corresponding author. Code Availability: Made available on request.
